# Methods for Monitoring Endoplasmic Reticulum Stress and the Unfolded Protein Response

**DOI:** 10.1155/2010/830307

**Published:** 2010-01-19

**Authors:** Afshin Samali, Una FitzGerald, Shane Deegan, Sanjeev Gupta

**Affiliations:** ^1^Department of Biochemistry, National University of Ireland, Galway, Galway, Ireland; ^2^National Centre for Biomedical Engineering Science, National University of Ireland, Galway, Galway, Ireland

## Abstract

The endoplasmic reticulum (ER) is the site of folding of membrane and secreted proteins in the cell. Physiological or pathological processes that disturb protein folding in the endoplasmic reticulum cause ER stress and activate a set of signaling pathways termed the Unfolded Protein Response (UPR). The UPR can promote cellular repair and sustained survival by reducing the load of unfolded proteins through upregulation of chaperones and global attenuation of protein synthesis. Research into ER stress and the UPR continues to grow at a rapid rate as many new investigators are entering the field. There are also many researchers not working directly on ER stress, but who wish to determine whether this response is activated in the system they are studying: thus, it is important to list a standard set of criteria for monitoring UPR in different model systems. Here, we discuss approaches that can be used by researchers to plan and interpret experiments aimed at evaluating whether the UPR and related processes are activated. We would like to emphasize that no individual assay is guaranteed to be the most appropriate one in every situation and strongly recommend the use of multiple assays to verify UPR activation.

## 1. Introduction

The endoplasmic reticulum (ER) is the cellular site for Ca^2+^ storage and for synthesis, folding, and maturation of most secreted and transmembrane proteins. Physiological or pathological processes that disturb protein folding in the endoplasmic reticulum cause ER stress and activate a set of signaling pathways termed the Unfolded Protein Response (UPR) [1]. This concerted and complex cellular response is mediated initially by three molecules, PKR-like ER kinase (PERK), activated transcription factor 6 (ATF6), and Inositol-requiring enzyme 1 (IRE1) [2]. The ER luminal domain of PERK, IRE1, and ATF6 interacts with the ER chaperone GRP78 (glucose-regulated protein); however, upon accumulation of unfolded proteins, GRP78 dissociates from these molecules, leading to their activation [3]. Notably, activation of ER stress sensors is modulated by other cellular factors, in addition to the dissociation of GRP78. A mutant of yeast IRE1, having deletion of GRP78 binding site in the ER luminal domain, is not constitutively active. Furthermore activation of this mutant (GRP78 binding site deleted) is regulated by accumulation of unfolded proteins in the ER [4, 5]. Dimerization of core stress-sensing region (CSSR) of the ER luminal domain of IRE1 creates a shared central groove similar to the peptide binding domains of major histocompatibility complexes (MHCs) [6–8]. It is proposed that MHC-like groove binds portions of unfolded polypeptide chain to promote formation of higher-order oligomers necessary for UPR activation [6–8]. Indeed luminal domain of yeast IRE1 interacts with unfolded proteins and inhibits aggregation of denatured proteins in vitro [7]. However, the ER luminal domain fragments of mammalian IRE1*α* did not interact with unfolded proteins in vitro [9]. IRE1 and PERK have conserved essential structural motifs in their ER luminal domains required for their dimerization. Similar to IRE1, ER luminal domain of PERK can also inhibit aggregation of denatured proteins in vitro [7]. Thus IRE1 and PERK appear to be regulated both by GRP78 and by direct binding of unfolded proteins. Activation of ATF6 is also regulated by combination of two discrete events: firstly by interaction with GRP78 and secondly by intra- and intermolecular disulfide bridges [10, 11]. The ER luminal region of ATF6 has two Golgi localization signals: GLS1 and GLS2. Binding of GRP78 masks the GLSs in the luminal domain of ATF6, and dissociation of GRP78 allows ATF6 to be transported to the Golgi body [11]. Further ER luminal domain of ATF6 is disulfide bonded and ER stress-induced reduction plays important role in both translocation to Golgi body and subsequent recognition by the site-1 and site-2 proteases (S1P and S2P) [10]. These differences may explain the different kinetics in the activation of IRE1, PERK, and ATF6 to various ER stress inducers.

Activated PERK phosphorylates translation initiation factor 2*α* (eIF2*α*), thereby reducing the rate of translation and the protein load on the ER [12, 13]. Phosphorylation of eIF2*α*  paradoxically increases translation of ATF4 mRNA to produce a transcription factor that activates expression of several UPR target genes [12, 14]. Activation of the ER protein kinase IRE1 triggers its endoribonuclease activity to induce cleavage of X box-binding protein 1 (XBP1) mRNA. XBP1 mRNA is then ligated by an uncharacterized RNA ligase and translated to produce spliced XBP1 protein [15]. Spliced XBP1 protein is a highly active transcription factor and one of the key regulators of ER folding capacity [16]. Concurrently, ATF6 is released from GRP78 and transits to the Golgi body where it is cleaved to release a transcriptionally active fragment [17]. Cleaved ATF6 acts in concert with spliced XBP1 protein to induce expression of genes encoding protein chaperones and components of the ER-associated degradation (ERAD) machinery [18, 19]. Moreover, ER stress can also induce autophagy [20], a catabolic cellular program that promotes cell survival in many contexts but which has been associated with induction of nonapoptotic cell death in others [21]. 

As discussed above the three proximal sensors of ER stress are PERK, ATF6, and IRE1. Exposure to ER stress activation of these proximal sensors leads to autophosphorylation of IRE1 at serine 724, autophosphorylation of PERK at threonine 980, and proteolytic processing of full-length ATF6 [1, 2]. The 90 kDa full-length ATF6 is processed within the Golgi body to its active 50 kDa form through sequential cleavage by site-1 and site-2 proteases (S1P and S2P) [17]. Therefore, proteolytic processing of ATF6 and phosphorylation of PERK and IRE1 can serve as markers of their activation status. However, detection of cleaved ATF6, phospho-PERK and phosho-IRE1 is quite difficult as these are expressed at very low levels and there is currently a lack of good commercial antibodies to detect them. Over the last 10 years, rapid progress has been made in understanding the molecular mechanisms of the UPR, and a number of genes modulated by the UPR have been identified. Most of these genes function in restoring ER homeostasis and alleviating ER stress. Therefore, these genes can be used as specific markers for the UPR. In our experience detection of the proteolytic processing of ATF6 or the phosphorylation of PERK and IRE1 is not advisable. Instead we suggest that detection of downstream protein targets of ER stress such as CHOP, HERP, XBP1, GRP78, and ATF4 (http://saturn.med.nyu.edu/research/mp/ronlab/Postings/UPR.detect.html) be a more robust approach for detecting activation of the UPR. One of the most commonly used indicators of ER stress is an increase in the expression level and the nuclear translocation of the transcription factor C/EBP homologous protein (CHOP) [22, 23]. However, it was recently reported that three out of seven commercially available CHOP antibodies gave false results by western blotting and immunocytochemistry for detection of CHOP [24]. Furthermore, there was a lot-to-lot variance in specificity from the same commercial source [24]. Therefore, we advise first validating the specificity of the antibody used for detecting CHOP protein expression to establish the presence or absence of ER stress. 

UPR pathways are important for normal cellular homeostasis and development and also play key roles in the pathogenesis of many diseases [25, 26]. Examples of pathophysiological conditions that can perturb the ER homeostasis include stroke, ischemia, diabetes, viral infections, and mutations that impair protein folding [25, 26]. Although the importance of ER stress and the UPR is being increasingly recognized, we still have only a limited number of good diagnostic methods to monitor the UPR. This limitation impedes our complete understanding and monitoring of the UPR, and in some cases, it may result in confusion. Importantly, there are no absolute criteria for determining the UPR signaling that can apply to every situation. This is because some assays are inappropriate, problematic, or may not work at all in particular cells, tissues, or model systems.

## 2. Experimental Approaches for the Detection of ER Stress

### 2.1. Splicing of XBP1 mRNA

**Figure 1:**
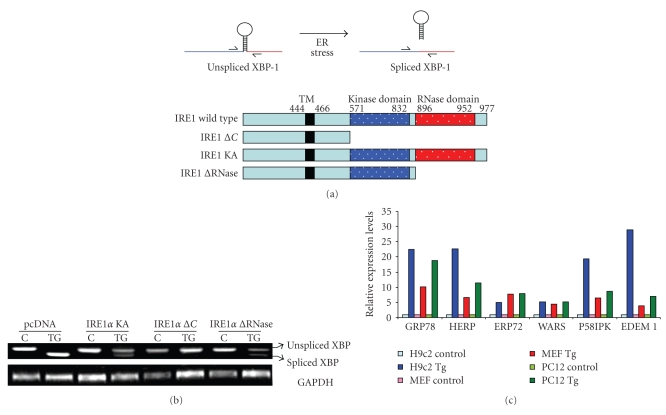
*Detection of transcript levels of UPR target genes by RT-PCR*. (a) Upper panel, cartoon of XBP1 splicing during ER stress. Lower panel, schematic representation of various mutant constructs of IRE1. (b) Modulation of XBP1 splicing by mutant IRE1. Total RNA was isolated from HEK 293 cells that were transfected with IRE1 mutants, either untreated or treated with thapsigargin (0.5 *μ*M) 6 hours, and RT-PCR analysis of total RNA was performed to simultaneously detect both spliced and unspliced XBP1 mRNA and GADPH. (c) Induction of UPR target genes upon exposure to thapsigargin. Total RNA was isolated from indicated cells after treatment thapsigargin (Tg), and the expression levels of the indicated genes were determined by real-time RT-PCR, normalizing against GAPDH expression.

In response to accumulation of unfolded proteins in the ER, IRE1 oligomerizes in the plane of the membrane, allowing for transautophosphorylation of juxtaposed kinase domains. The transautophosphorylation of the kinase domain of IRE1 activates its unusual effector function, which causes the unconventional splicing of the mRNA that encodes a transcription factor named XBP1 [15]. In metazoans, a 26-nucleotide intron is spliced out by activated IRE1, leading to a shift in the codon reading frame (Figure 1(a)). The XBP1 protein encoded by the spliced mRNA is more stable and is a potent transcription factor of the basic-leucine zipper (bZIP) family and one of the key regulators of ER folding capacity [15, 16]. The splicing of XBP1 mRNA can be detected by semiquantitative RT-PCR using primers specific for *XBP1* which will detect both unspliced and spliced isoforms. The 5′ to 3′ sequences of primers used to detect unspliced and spliced XBP1 mRNA are as indicated below.

**Rat XBP1**

Forward primer: TTACGAGAGAAAACTCATGGGC

Reverse primer: GGGTCCAACTTGTCCAGAATGC

Size of PCR products: unspliced XBP1 = 289 bp, spliced XBP1 = 263 bp.

**Human XBP1**

Forward primer: TTACGAGAGAAAACTCATGGCC

Reverse primer: GGGTCCAAGTTGTCCAGAATGC

Size of PCR products: unspliced XBP1 = 289 bp, spliced XBP1 = 263 bp.

**Mouse XBP1**

Forward primer: GAACCAGGAGTTAAGAACACG

Reverse primer: AGGCAACAGTGTCAGAGTCC

Size of PCR products: unspliced XBP1 = 205 bp, spliced XBP1 = 179 bp.

We have detected IRE1-dependent splicing of XBP1 mRNA under conditions of ER stress by using various mutants of IRE1 (Figures 1(a) and 1(b)). A variety of mammalian cell lines can be used to determine the splicing of XBP1. To follow this method, cells should be seeded on six-well plates and transfected with indicated IRE1 mutants. Twenty-four hours post transfection, cells are subjected to ER stress stimuli, for example, tunicamycin, thapsigargin, or Brefeldin A for different time points ranging from 6–48 hours. Three chemicals are generally used to experimentally induce ER stress: tunicamycin (Sigma), thapsigargin (Sigma), and Brefeldin A (BFA) (Sigma). Although these chemicals target different components of the ER, their common effect is to interfere with ER functions and thereby lead to ER protein misfolding. Tunicamycin inhibits N-linked glycosylation, while thapsigargin blocks the ER calcium ATPase pump, leading to the depletion of ER calcium stores. Brefeldin A interferes with protein transport from the endoplasmic reticulum to the Golgi apparatus by inhibiting transport in the Golgi, which leads to proteins accumulating inside the ER. The concentration and time of treatment depend on system being studied and need to be determined individually for each system. Cells are harvested and total RNA is isolated using RNeasy kit (Qiagen) or TRIzol reagent (Invitrogen) according to the manufacturer's instructions. Reverse transcription (RT) is carried out with 2 *μ*g RNA and Oligo dT (Invitrogen) using 20 U Superscript II reverse transcriptase (Invitrogen). Then standard conditions of RT-PCR can be used to determine the unspliced and spliced isoforms of XBP1 (Figure 1(b)). The ER stress-mediated splicing of XBP1 requires activation of IRE1, and if the function of IRE1 is compromised, ER stress-mediated splicing of XBP1 is attenuated (Figure 1(b)).

### 2.2. mRNA Levels of UPR Target Genes

The ER stress response is an autoregulatory program that upregulates a large number of genes that expand the folding capacity of the ER, such as ER chaperones and ERAD components [1]. Mapping of the promoters of a number of ER stress responsive genes, such as BiP/GRP78, GRP94, calreticulin, HERP, EDEM1, and HRD1, have identified three cis-acting response elements, namely, ERSE (ER Stress Response Element), ERSE-II (ER Stress Response Element II), and UPRE (Unfolded Protein Response Element) [27–31]. ERSE has a consensus sequence CCAAT-N9-CCACG, which is necessary and sufficient for the induction of at least three major ER chaperones (GRP78, GRP94, and calreticulin) [28, 31]. HERP, one of the most highly inducible genes during the UPR, has a promoter that contains not only ERSE but also a cis*-*acting element with a sequence of ATTGG-N_1_-CCACG termed ESRE-II [27]. UPRE which contains the consensus sequence TGACGTGG/A was originally identified as a DNA sequence bound by bacterially expressed ATF6 [29]. Loss of ATF6 leads to reduced activation of UPRE containing genes such as EDEM1 and HRD1 [19]. We recommend determining the transcript levels of bona fide UPR target genes whose induction has been reported to occur during conditions of ER stress and whose promoter regions contain at least one of the three cis-acting response elements, namely, ERSE, UPRE, or ERSE-II. 

In our laboratory, the induction of mRNA of UPR target genes has been detected in a variety of mammalian cell lines using real-time RT-PCR (Figure 1(c)). Cells were generally induced to undergo ER stress by incubating with tunicamycin, thapsigargin, or Brefeldin A. The concentration and time of treatment depend on system being studied and need to be determined individually for each system. In these experiments cells were treated with ER stress inducing agents such as Tg, Tm, and BFA and total RNA was isolated using RNeasy kit (Qiagen) or TRIzol reagent (Invitrogen) according to the manufacturer's instructions. Reverse transcription (RT) was carried out with 2 *μ*g RNA and Oligo dT (Invitrogen) using 20 U Superscript II Reverse Transcriptase (Invitrogen). For real-time PCR experiments, cDNA products were mixed with 2 × TaqMan master mixes and 20 × TaqMan Gene Expression Assays (Applied Biosystems) and subjected to 40 cycles of PCR in StepOnePlus instrument (Applied Biosystems). Relative expression was evaluated with ΔΔCT method. We would like to point out that other methods for detection of mRNA levels such as northern blotting, RNAse protection assays, and conventional RT-PCR can also be used. We prefer real-time RT-PCR with TaqMan chemistry (also known as “fluorogenic 5′ nuclease chemistry”) because of its sensitivity, specificity, speed, and ease of handling. Table 1 provides a list of TaqMan Assays (Applied Biosystems) that have worked reproducibly in our experience to detect the transcripts of the several UPR markers.

### 2.3. Western Blotting and Immunohistochemistry for UPR Target Genes

**Figure 2:**
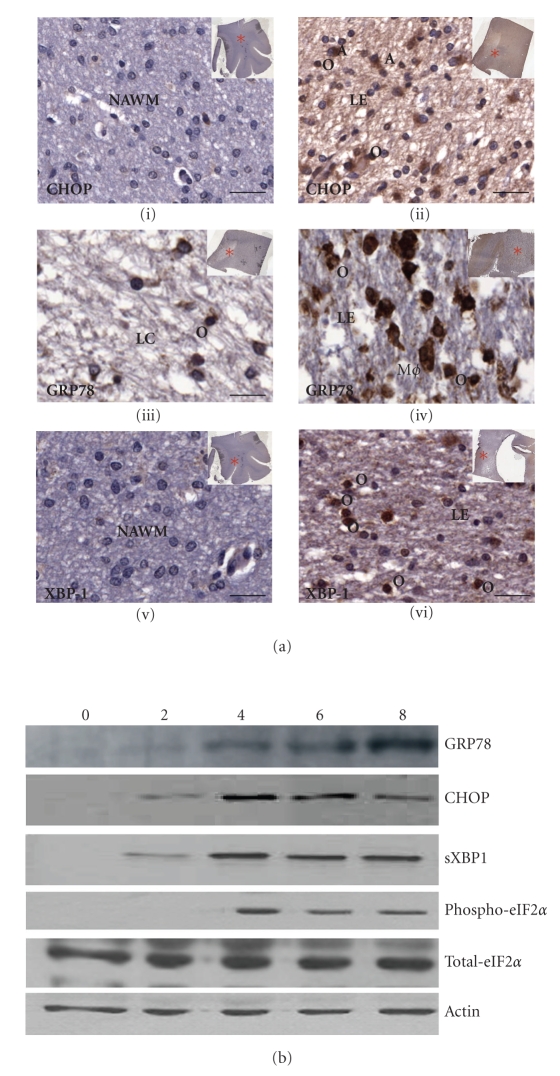
*Detection of protein levels of UPR target genes.* (a) Immunohistochemical detection of CHOP, GRP78, and XBP1 in Multiple Sclerosis patient postmortem brain tissue. Representative images showing upregulation of CHOP (ii) at the edge (LE) of a chronic active lesion, in comparison to (i) NAWM. GRP78 expression was downregulated in the center of a chronic active lesion (iii) when compared to the edge (iv) of actively demyelinating lesions. Sample images illustrate the variety of morphologically distinct cell types that express CHOP or GRP78 including macrophages (Mø), astrocytes (a), and oligodendrocytes (o). Increased expression of XBP1 was found at the edge of a chronic active lesion (vi), when compared to normal-appearing white matter (v). XBP1 immunostaining is also apparent in a large number of oligodendrocytes (o). All immunoperoxidase-stained cells were detected using the chromogen, DAB (brown), and counterstained with hematoxylin for nuclei (blue). Scale bars = 250 *μ*m. Letter codes are as follows: NAWM = normal-appearing white matter; LC = lesion center; LE = lesion edge. Red astrices indicate location of lesion within brain sections analyzed. (b) PC12 cells were treated with 0.25 *μ*M of Tg for 0, 2, 4, 6, and 8 hours. Whole cell lysates were analyzed by Western Blot for GRP78, CHOP, spliced XBP1, phospho-eIF-2*α*, and total- eIF-2*α*. *β*-Actin was used to determine equal loading of samples.

We recommend determining the protein levels of established UPR target genes whose induction has been reported to occur during relevant conditions of ER stress. Activation of the UPR has been found in various pathological states of the brain including ischemia and degenerative diseases. Increased phosphorylation of PERK has been shown after cerebral Ischemia and reperfusion by immunohistochemical analysis [32]. Several postmortem studies of primary human Alzheimer's disease brain tissues show evidence of ER stress in the form of enhanced ER chaperone expression and immunohistochemical reactivity for specific markers of the UPR [33, 34]. Recently we found increased expression of GRP78, CHOP, and XBP1 in acute, active, and chronic multiple sclerosis (MS) lesions by immunohistochemical and dual-immunofluorescent analyses [35]. Figure 2(a) shows immunohistochemical staining of fixed frozen paraffin-embedded (FFPE) brain tissue sections from MS patient which showed upregulation of CHOP, GRP78, and XBP1. Specific antibodies used are detailed in Table 1. FFPE tissue was used in preference to frozen blocks as in our hands it yielded higher quality staining with lower background and fewer staining artifacts. Following deparaffinization, all sections were incubated for 10 minutes at room temperature in 3% hydrogen peroxide in methanol (Sigma-Aldrich, Dublin), to block endogenous peroxidases. For CHOP and GRP78 staining, antigen retrieval was achieved by incubating sections in 0.01 M Tris-EDTA pH 9 (Sigma-Aldrich, Dublin) for 2 minutes in a Tefal pressure cooker at full steam. To retrieve antigen before XBP1 staining, tissue was placed in 0.01 M citrate pH 9 (Sigma-Aldrich, Dublin) before microwaving it for 20 minutes in a 700 watt Sanyo microwave. Bound CHOP, GRP78, or XBP1 antibody was detected following incubation for 30 minutes at room temperature in peroxidase-labeled EnVision anti-mouse or anti-rabbit antibody (Dako, Ely, UK) with 3,3′- diaminobenzidine (DAB) as a chromogen (Dako, Ely, UK). 

When carrying out western blotting, we suggest performing standard procedures to determine the protein levels of bona fide UPR target genes within protein samples (Figure 2(b)). Table 2 provides a list of antibodies that have worked best and most reproducibly in our experience to detect several UPR marker proteins in western blotting and immunohistochemistry.

### 2.4. Reporter Assays for Activity of XBP1 and ATF6

The most salient feature of the UPR is an increase in the transactivation function of a number of bZIP transcription factors, such as ATF6, ATF4, and XBP1. It has been well established that transcriptional induction of UPR target genes upon ER stress is mediated by the cis-acting response elements. There are several reporter systems which can be used to detect ATF6 and XBP1 activation. In the p5xATF6-GL3 reporter, the luciferase gene is under the control of the c-fos minimal promoter and five tandem copies of the ATF6 consensus binding site identifed by in vitro gel mobility shift assays with recombinant ATF6 [29]. In p4xXBPGL3 reporter, the luciferase gene is under the control of four tandem copies of the XBP1 consensus binding site 5′-CGCG(TGGATGACGTGTACA)_4_-3′ [16]. In addition there are several other ERSE reporters which have promoter regions of GRP78, GRP94, Calreticulin, XBP1 [28], and an ERSE-II reporter which has the HERP promoter upstream of the luciferase reporter gene [27]. These reporters should be used in combination with the corresponding mutant promoter where the functional cis-elements have been mutated. The advantage of these reporters is that they can be used to monitor the activation of endogenous ER stress. However, there is some question as to whether these reporters respond primarily to endogenous ATF6 and/or XBP1, since XBP1's binding site is similar to the ATF6 site, and activated forms of both ATF6 and XBP1 can activate the reporter. Furthermore, ATF6 and XBP1 can heterodimerize in vivo and ATF6-XBP1 heterodimer possesses 8-fold higher affinity for the UPRE than that for XBP1 homodimer [19]. Nevertheless, luciferase-based reporters are a very sensitive method to detect ER stress whether it measures activation of ATF6, XBP1, or both.

A variety of mammalian cell lines can be used to determine the activity of XBP1/ATF6 using these reporter constructs. Cells should be seeded on six-well plates and transfected by the optimized transfection method 24 hours later. The transfection mixture for each well should contain the luciferase reporter gene and an internal control to normalize the transfection efficiencies (Renilla luciferase or *β*-galactosidase). The internal control plasmid is not responsive to ER stress. 24 hours post transfection, cells are induced to undergo ER stress by incubating with appropriate concentrations of tunicamycin, thapsigargin, or Brefeldin A for different time points ranging from 6–48 hours. Cells are then harvested and the firefly luciferase present in the cell lysate is measured along with the appropriate internal control (Renilla luciferase or *β*-galactosidase). The results should be normalized to the internal control for each point to determine the fold induction in the reporter activity.

### 2.5. Detection of IRE1 Activation and ATF6 Translocation from the ER to the Nucleus with Fluorescent Microscopy

**Figure 3:**
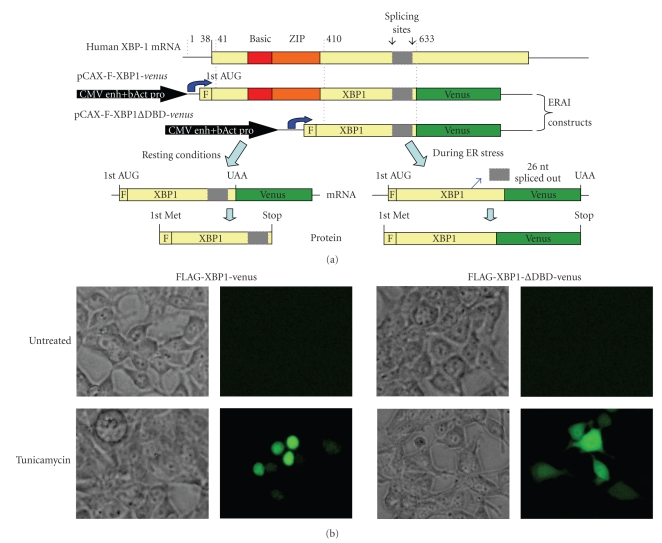
*Detection of IRE1 activity using “ER stress-activated indicator” (ERAI) constructs.* (a) Schematic presentation of ERAI plasmid obtained by fusing XBP1 and *venus*, a variant of the green fluorescent protein (adapted from [36] by Iwawaki et al. (2004)). (b) Twenty-four hours after transfection F-XBP1-*venus* and F-XBP1ΔDBD-*venus*, 293T cells were left untreated or treated with (1 *μ*g/mL) tunicamycin for 24 hours and then analyzed by fluorescence microscopy.

ER stress-dependent splicing of XBP1 has been used to develop fluorescent reporter constructs by fusing XBP1 sequence to *venus*, a variant of green fluorescent protein which enables the activation of IRE1 to be monitored [36, 37]. The design of the XBP1-*venus* reporter is shown in Figure 3(a). In this construct, the gene encoding *venus* is cloned downstream the 26-nt ER stress-specific intron of human *XBP1 *[36]. Under normal conditions, the mRNA of the fusion gene is not spliced, and its translation terminates at the stop codon near the joint between the *XBP1* and *venus* genes. However, during ER stress, the 26-nt intron is spliced out, leading to a frame shift of the chimeric XBP1-*venus* mRNA, similar to that of the endogenous XBP1 mRNA. Translation of the spliced mRNA produces an XBP1-*venus* fusion protein and cells experiencing ER stress can be detected by monitoring the fluorescence activity of *venus*. As *venus* expression can only occur from the spliced form of the XBP1-GFP mRNA, its presence signals the activation of IRE1. Upon transfection of the XBP1-GFP reporter into cells, tunicamycin treatment results in detectable fluorescence in the nucleus, whereas negligible fluorescence is detected in any compartment under normal conditions [36, 37]. Moreover, *venus* expression during tunicamycin treatment has been shown in splicing assays to correlate with the extent of splicing of the UPR intron from XBP1/GFP mRNA [36, 37]. We have used 293T cells to detect activation of IRE1 using two different XBP1-*venus* reporter plasmids: F-XBP1-*venus* and F-XBP1ΔDBD-*venus* (Figure 3(a)). In F-XBP1ΔDBD-*venus* construct, DNA-binding domain (DBD) of XBP1 is deleted. F-XBP1ΔDBD-*venus* construct is recommended for use as overexpression of F-XBP1ΔDBD-*venus* does not affect induction of UPR target genes and can be used to detect activation of IRE1 similar to F-XBP1-*venus* construct. F-XBP1ΔDBD-*venus* construct has been used to generate a transgenic mouse model for monitoring ER stress (discussed later). Twenty-four hours post transfection, cells are induced to undergo ER stress by incubating with appropriate concentrations of tunicamycin, for 24 hours. In the cells transfected with F-XBP1-*venus* construct, tunicamycin treatment leads to appearance of green fluorescence in the nucleus (Figure 3(b)). However in the cells transfected with F-XBP1ΔDBD-*venus* construct, tunicamycin treatment leads to appearance of green fluorescence in the cytosol (Figure 3(b)). One important point to note is that overexpression of F-XBP1-*venus* construct interferes with induction of UPR target genes in a dominant-negative manner [36]. The major drawback, however, is the relatively large amount of GFP that needs to be expressed in the cell for visualization by microscopy. Thus, there will be a time lag between actual IRE1 activation and its detection by the accumulation of GFP.

**Figure 4:**
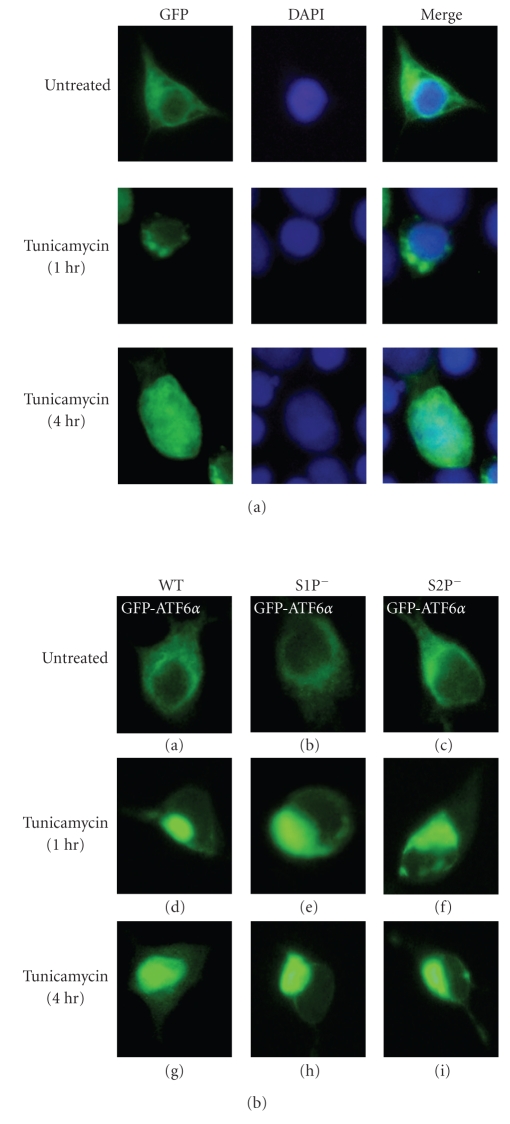
*ER stress-induced processing and nuclear translocation of GFP-ATF6.* (a) Twenty-four hours after transfection with pCMVshort-EGFP-ATF6 (WT), 293T cells were left untreated or treated with 1 *μ*g/mL tunicamycin for the indicated periods. Cells were fixed in 4% paraformaldehyde, stained with DAPI, and then analyzed by fluorescence microscopy. (b) Twenty-four hours after transfection with pCMVshort-EGFP-ATF6*α*(WT), pCMVshort-EGFP-ATF6*α*(S1P^−^), or pCMVshort-EGFP-ATF6*α*(S2P^−^), 293T cells were left untreated or treated with 1 *μ*g/mL tunicamycin for the indicated periods and then analyzed by fluorescence microscopy.

A key regulatory step in ATF6 activation is its transport from the ER to the Golgi body, where it is processed by S1P and S2P proteases [38, 39]. The cytoplasmic fragment of ATF6, thereby liberated from the membrane, translocates into the nucleus and activates transcription of its target genes [38, 39]. A GFP-ATF6 fusion protein, which relocates from the ER to the nucleus via the Golgi apparatus in response to ER stress, can be used to monitor activation of ATF6 by fluorescent microscopy [38, 39]. One limitation of this approach, however, is that overexpression can sometimes alter the subcellular localization and kinetics of protein trafficking. This problem has been addressed to some extent by expressing GFP-ATF6 from a shortened CMV promoter which has a deletion of 430 base pairs from the 5′ side. The short promoter possesses considerably lower activity than the full promoter and GFP-ATF6 expressed using the short CMV promoter is localized exclusively to the ER and translocates to the nucleus similarly to endogenous ATF6 [39]. For detection of GFP-ATF6, 293T cells were transfected with pCMVshort-EGFP-ATF6 (WT), pCMVshort-EGFP-ATF6 (S1P^−^), and pCMVshort-EGFP-ATF6 (S2P^−^) plasmids. pCMVshort-EGFP-ATF6 (S1P^−^) and pCMVshort-EGFP-ATF6 (S2P^−^) have a mutation that abrogates the cleavage by S1P or S2P, respectively. 24 hours post transfection, cells were treated with 1 *μ*g/mL tunicamycin. As shown in Figure 4(a), the wild-type GFP-ATF6 was translocated to the nucleus via the Golgi apparatus. Both EGFP-ATF6 (S1P^−^) and EGFP-ATF6 (S2P^−^) were localized in 293T cells similarly to the wild-type GFP-ATF6 (Figure 4(b): a–c). In contrast to wild-type GFP-ATF6 (Figure 4(b): a, d, g), GFP-ATF6_(S1P^−^) (Figure 4(b): b, e, h) and EGFP-ATF6 (S2P^−^) (Figure 4(b): c, f, i) remained associated with the Golgi apparatus even 4 hours after tunicamycin treatment. These results demonstrate that cleavage by S1P and S2P is critical for the processing of GFP-ATF6 and that only the processed product, GFP-ATF6, can enter the nucleus. The advantage of GFP is that its intrinsic fluorescence allows the translocation of ATF6 to be continuously followed in single living cells and the whole process recorded over time using, for example, time-lapse photography. 

### 2.6. Use of Transgenic Models

ER stress has been implicated in human neuronal diseases, such as Parkinson's disease, Alzheimer's disease, as well as other disorders [25]. The exact contributions to and casual effects of ER stress in the various disease processes are not known. Furthermore, components of ER stress signaling are also required during development [40, 41]. Studies of ER stress in vivo will provide information that is important and useful in pathology and developmental biology. Two different transgenic mouse models have been described for monitoring ER stress in vivo. The first model, referred to as “ER stress-activated indicator” (ERAI), was constructed by fusing XBP1 and *venus*, a variant of the green fluorescent protein (described in Section 2.4) [36]. This mouse model could serve as a specific and sensitive indicator of ER stress in vivo during development and disease, as well as for analysis of drug effects on ER function. However, this ERAI model detects activation of IRE1 only and does not reveal any information about ATF6 and PERK activation. The other limitations of this model include lack of ERAI expression in some cell types and the inability to detect weak ER stress signals.

The second model, known as ERSE-LacZ model, was constructed by using a LacZ reporter gene driven by 3 kilobases of the rat GRP78 promoter [42]. Two additional transgenic lines have been reported for this model. First, the D300LacZ mouse contains a 230 bp internal deletion spanning from −300 to −70, which eliminates the known ER stress-inducible elements of the GRP78 promoter, including both the ERSE and the cAMP-response element (CRE) [42]. Second, the D170LacZ mouse has a 100 bp internal deletion spanning −170 to −70, which eliminates only the three tandem copies of the ERSE [42]. The wild-type ERSE-LacZ model recapitulates the endogenous expression profile of GRP78 with highest expression in the early embryonic heart which is dependent on the presence of ERSE in the promoter region of GRP78. When using the ERSE-LacZ model, it is recommended to use wild-type GRP78 promoter along with ERSE-deleted GRP78 promoter. ERSE-deleted GRP78 promoter serves as an important control for specificity of ERSE-mediated ER stress in vivo. However, this system does not reveal any information about the three different arms of UPR. One obvious limitation of the ERSE-LacZ model is possible interference by signals not directly related to ER stress since expression of GRP78 is regulated by the coordinated function of several other transcription factors that can act outside of ERSE. Therefore, while both ERAI and ERSE-lacZ mouse models have their unique advantages and pitfalls, they may complement each other to provide novel insights into the complexity of ER stress signaling in vivo in multicellular organisms.

## 3. Concluding Remarks

In addition to maintaining the homeostasis of ER function, the ER stress response is involved in a number of cellular processes. It has been shown that ER stress is induced during the differentiation of B cells into antibody-secreting plasma cells, likely due to the need to increase the secretory capacity of the cells. In addition, ER stress activation is associated with several human diseases including Alzheimer's disease, diabetes, and atherosclerosis. The experimental approaches discussed above should prove useful to those researching ER stress in vitro and in vivo. Further, these experimental strategies may evolve as new methodologies are developed and our understanding of UPR improves. Nonetheless, it is useful to establish guidelines for acceptable assays that can reliably monitor UPR in many experimental systems. 

## Figures and Tables

**Table 1:** List of TaqMan assays that reproducibly detect markers of UPR.

	Rattus norvegicus		Mus musculus	
Target gene	Accession number	Assay number	Accession number	Assay number
GRP78	NM_013083.1	Rn01435771_g1	NM_022310.2	Mm01333324_g1
HERP	NM_053523.1	Rn01536690_m1	NM_022331.1	Mm01249592_m1
ERP72	NM_053849.1	Rn01451754_m1	NM_009787.2	Mm00437958_m1
WARS	NM_001013170.2	Rn01429998_g1	NM_011710.2	Mm00457097_m1
P58IPK	NM_022232	Rn00573712_m1	NM_008929	Mm00515299_m1
EDEM1	XM_238366.4	Rn01765441_m1	NM_138677.2	Mm00551797_m1

**Table 2:** List of antibodies that reproducibly detect markers of UPR.

Target name	Supplier	Applications
phospho-PERK	#3191; Cell Signaling	WB (1 : 2000), IHC (1 : 100)
phospho-PERK	#3179; Cell Signaling	WB
CHOP	MA1-250, Affinity bioreagents	WB
CHOP	sc-793; Santa Cruz Biotechnology	WB (1 : 1000)
		IHC (1 : 400–1 : 800)
spliced XBP-1	sc-7160;Santa Cruz Biotechnology	WB (1 : 2000)
		IHC (1 : 100)
ATF4	ARP37017_P050; Aviva Systems Biology	WB (1 : 5000)
Grp78	SPA-926; Stressgen	WB (1:1000)
	AB32618; Abcam	IHC (1 : 200)
phospho-eIF2 alpha	#9721; Cell Signaling	WB (1 : 2500), IHC (1 : 100)
total-PERK antibody	sc-9477; Santa Cruz Biotechnology	WB (1 : 1000), IP
IRE1-alpha	#3294; Cell Signaling	WB (1 : 1000)

## Abbreviations

ATF4: Activating transcription factor 4
ATF6: Activating transcription factor 6
BiP: Binding immunoglobulin protein
BFA: Brefeldin A
bZIP: Basic leucine zipper domain
CHOP: CAAT/enhancer binding protein (C/EBP) homologous protein
CMV: Cytomegalovirus
CRE: cAMP-response element
eIF2*α*: eukaryotic initiation factor 2*α*
ERAD: ER-associated degradation machinery
EDEM1: ER degradation enhancer, mannosidase alpha-like 1
ERSE: ER stress response element
ERAI: ER stress-activated indicator
FFPE: Fixed frozen paraffin-embedded
GRP78: glucose regulated protein 78
GRP94: glucose-related protein 94
GFP: Green fluorescent protein
HERP: homocysteine-induced ER protein
HRD1: HMG-coA reductase degradation 1
IRE1: Inositol-requiring enzyme 1
MS: Multiple sclerosis
PCR: Polymerase chain reaction
PERK: PKR-like ER kinase
RT: Reverse transcription
S1P: Site-1 protease
S2P: Site-2 protease
TG: Thapsigargin
TM: Tunicamycin
UPR: Unfolded protein response.
